# Supplementary material for: Specific Antibodies and Arachidonic Acid Mediate the Protection Induced by the Schistosoma mansoni Cysteine Peptidase-Based Vaccine in Mice
Source: Vaccines (Basel). 2020 Nov 16;8(4):682. doi: 10.3390/vaccines8040682 (PMC7712720; doi:10.3390/vaccines8040682)
Supplement: Supplementary file 1 [file vaccines-08-00682-s001.pdf]

Table S1. Effect of immunization with SmCB1 or SmCL3 on worm burden of challenge *S. mansoni* infection in outbred mice.

		VACCINE	
**PARAMETER**	Infected controls	SmCB1-immunized	SmCL3-immunized
**Individual worm burden**			
	**54**	28	30
	**52**	22	32
	**53**	23	28
	**58**	20	27
	**58**	25	25
	**62**	27	31
	**67**	26	25
	**60**	24	29
**Mean**	**58.0**	24.3	28.3
**SD**	**5.1**	2.7	2.6
**Percent reduction**		58.1	51.2
***p* value**		0.0002	0.0002

Vaccinated mice were challenged 3 weeks after second immunization with 150 cercariae of *S. mansoni* in parallel with unimmunized mice (infected controls), and assessed (eight per group) for worm burden 40 days post infection. ANOVA, Students' –*t*- 2-tailed and Mann-Whitney tests were used to analyze the statistical significance of differences between selected values and considered significant at $P < 0.05$. Significances of differences between immunized and unimmunized control mice are shown.

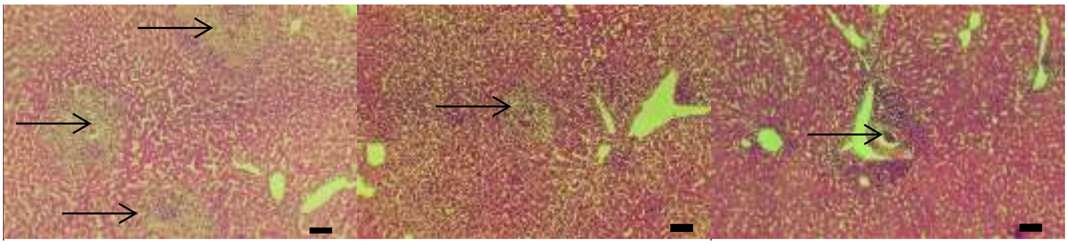

a b c

Figure S1. Haematoxylin and eosin-stained liver sections of unimmunized infected (infected), and SmCB1 and SmCL3-immunized mice 40 days post infection. Arrows point to circumoval granulomas. Scale bars = 30 µm.a: Infected, b: SmCB1, c: SmCL3

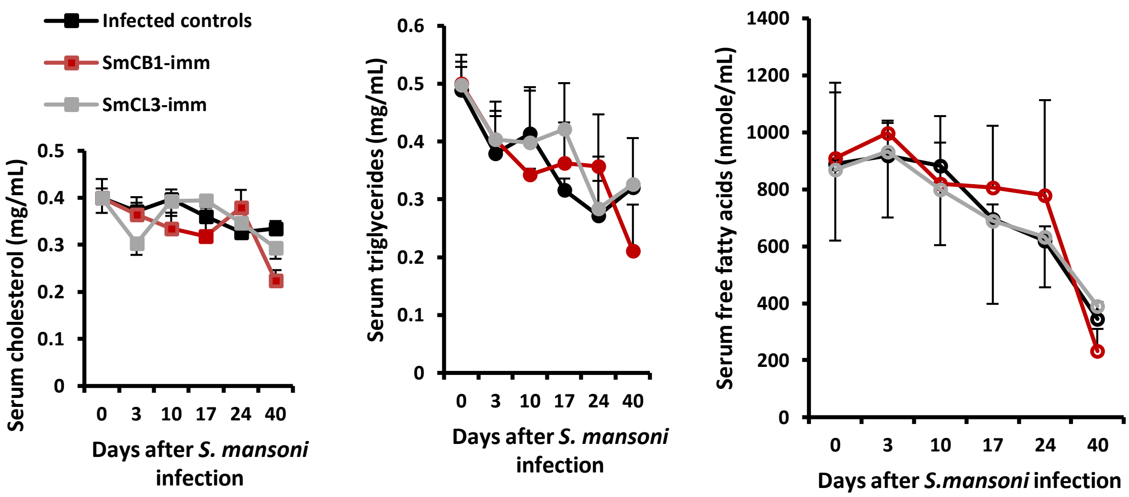

Figure S2. Each point in the figures represents mean of serum lipid values of 6 individual mice. Naïve control mice had 0.4 ± 0.1 mg/mL serum cholesterol, 0.48 ± 0.2 mg/mL serum triglycerides, and 800 ± 120 nmol free fatty acids/mL.
